# A High-Throughput ImmunoHistoFluorescence (IHF) Method for Sub-Nuclear Protein Analysis in Tissue

**DOI:** 10.3390/cells14141109

**Published:** 2025-07-18

**Authors:** Kezia Catharina Oxe, Kristoffer Staal Rohrberg, Ulrik Lassen, Dorthe Helena Larsen

**Affiliations:** 1 Nucleolar Stress and Disease Group, Danish Cancer Institute, Danish Cancer Society, 2100 Copenhagen, Denmark; 2 Department of Oncology, Rigshospitalet University Hospital of Copenhagen, 2100 Copenhagen, Denmark

**Keywords:** ImmunoHistoFluorescence, sub-nuclear protein analysis, tissue analysis

## Abstract

The current understanding of cellular protein distribution in clinical samples is limited. This is partially due to the complexity and heterogeneity of tissues combined with the qualitative nature of analysis by immunohistochemistry (IHC). The common use of manual assessment in the clinic is time-consuming and restricts both the complexity of scoring and the scale of patient tissue analysis. This has limited the transfer of biological observations into pathology and their integration into diagnostics. Immunofluorescence (IF) techniques allow detailed and high-throughput investigation of proteins in cell models, but their application to tissues has been hindered by poor antibody penetration, autofluorescence artefacts, and weak signals. With a growing focus on precision medicine, scalable techniques to investigate and analyse proteins are critically important. To address this, we generated a high-throughput ImmunoHistoFluorescence (IHF) approach, applying IF to tissue samples followed by automated acquisition and artificial intelligence (AI)-based analysis of sub-nuclear protein distribution to enable precise investigation of complex protein localization patterns. This advancement offers a method to transfer in vitro findings into human tissues to analyse protein localization patterns in physiologically relevant contexts for improved understanding of disease-driving mechanisms in patients, identification of new biomarkers, and acceleration of translational research.

## 1. Introduction

The understanding of biological mechanisms in human pathophysiology is more relevant than ever with the growing use of precision medicine, in particular in cancer treatment. For decades, immunohistochemistry (IHC) has served as the golden standard for tissue analysis in the clinic [1,2,3]. However, this approach often falls short in providing detailed cellular information of biological and clinical relevance. This can be attributed to not only the qualitative assessment of tissue samples but also manual microscopy techniques and analysis that limit the throughput and the possibilities for quantifications [4,5]. Furthermore, inter-observer consistency issues further limit the standardization of IHC analysis [6,7] that is required for the interpretation of biological phenotypes. Recently, the development of immunofluorescent multiplexing approaches for human tissues has addressed some of these limitations, enabling simultaneous detection of multiple proteins in a single tissue section, offering new opportunities for tissue profiling and spatial analysis [8,9]. So far, however, it remains challenging to transfer cell-based findings to tissues and thereby into a disease context.

In cell models, sub-cellular protein distribution and posttranslational modifications are widely studied using immunofluorescence (IF) techniques [10,11] combined with automated imaging systems [12]. Objective data acquisition and standardized automated data analysis have been enabled by advancements in hardware and software, including machine learning and artificial intelligence (AI) [13,14]. However, the implementation of IF for tissue samples has previously been unsuccessful due to limited antibody penetration, autofluorescence, and weak antigen expression that resulted in faint signals [15]. The complexity of cell-based data analysis achieved by automization [16] is also not transferrable to a manual setup, and its application to tissue samples remains limited [17,18].

Here, we present a method that combines IF, automated microscopy, and AI-based image analysis to allow the transfer of cell-based observations into tissues. To enable precise analysis of protein distributions and localizations in tissue samples at scale, we developed a high-throughput automated analysis scoring protein distribution patterns in a sub-nuclear compartment, in this paper the nucleolus. To accomplish this, we generated formalin-fixed paraffin-embedded (FFPE) cell blocks generated from human osteosarcoma cells, U2OS, with and without ribosomal DNA (rDNA) damage providing positive and negative controls for staining optimization and automation of the analysis. Moreover, we verified and applied our ImmunoHistoFluorescence (IHF) technique and automated acquisition on multi-organ tissue samples to demonstrate its broad applicability to different tissues, including specimens from both healthy and sick individuals. This IHF method provides a high-throughput system for precise detection of sub-nuclear protein distribution and localization in tissue.

## 2. Materials and Methods

### 2.1. Cell Culture

The wild-type (WT) human osteosarcoma cell line, U2OS, purchased from ATCC (HTB-96), was used in this study. U2OS cells were grown in Dulbecco’s modified Eagle’s medium (DMEM) with GlutaMAX^TM^ supplement. The medium was complemented with 10% foetal bovine serum (FBS) and 10 U penicillin-streptomycin (pen-strep). Cells were incubated at 37 °C with 5% CO_2_. The reagents used for cell culture are listed in the source data in Appendix A.

### 2.2. Ribonucleoprotein (RNP) Complex Transfection

Double-stranded breaks (DSBs) in the rDNA were induced by transfection of RNP complexes, consisting of recombinant Cas9 protein (TrueCut Cas9 protein v2) and sgRNAs. Transfections were performed using the Lipofectamine CRISPRMAX Cas9 transfection reagent according to the manufacturer’s protocol with some alterations to the protocol. For the transfections, only 25% of the recommended amount of Cas9, sgRNA, and Cas9 PLUS reagent were used, keeping the stoichiometric ratio as recommended. For induction of DSBs in the nucleolus, a combination of two sgRNAs (sgRNA1 and sgRNA3), targeting the rDNA [19,20] was used in a 1:1 molar ratio. sgRNA sequences [19] are listed in Supplementary Information. The reagents used for RNP complex transfection are listed in the source data in Appendix A.

### 2.3. Preparation of FFPE Cell Blocks

Following 8 h of RNP complex transfection, U2OS cells were harvested by scraping. The spun-down cell pellet was subsequently fixed in 4% formaldehyde for 24 h at room temperature (RT). The pellet was stored in 70% ethanol prior to histoprocessing. The cell pellet was processed in the Thermo Microm STP 120 Tissue Processor (Thermo Fisher Scientific, Vedbæk, Denmark) using the same SOP as used for tissues (explained below). The cell pellet was dehydrated through submergence in increasing concentrations of ethanol and subsequently cleared in xylene. Lastly, the pellet was embedded in paraffin in the CellSafe plus biopsy cassettes. The paraffin blocks were stored at RT prior to being sliced into 4 µm sections and mounted onto Vectabond-coated SuperFrost Plus slides. The reagents used for the preparation of the FFPE cell blocks are listed in the source data in Appendix A.

### 2.4. Preparation of TMA

The multi-organ TMA (Pantomics, Inc., #UNC241) used for this study was purchased through Pantomics, Inc., Fairfield, CT, USA (now QuickArrays). Human tissue samples acquired through Pantomics Inc., Fairfield, CT, USA were collected under strict IRB/HIPAA-approved guidelines with informed consents from the donors or their relatives. The rights to hold research uses for any purpose or further commercialized uses have been waived. Personal information for the tissue microarray is anonymized. The TMA used for IHF is listed in the source data in the Appendix A.

Pantomics, Inc., Fairfield, CT, USA describe their tissue processing as follows. All the tissues were fixed in 10% neutral buffered formalin for 24 h within 30 min of surgical removal and processed using identical SOPs commonly used in pathology departments throughout Europe and America. All FFPE sections were sliced 4 µm thick and were mounted onto SuperFrost Plus or APES-coated SuperFrost slides. The TMA was stored at 4 °C prior to IHF staining.

### 2.5. ImmunoHistoFluorescence Staining

Paraffin on slides with FFPE tissues was melted by heating the slides vertically at 60 °C for 20 min. The tissues were further de-paraffinized through submersion in cold xylene for 15 min and rehydrated for 2 min in decreasing concentrations of ethanol using a serial dilution row ranging from 100%, 96%, 80%, 70% to 50%. To restore epitope masking, the slides were submerged in antigen retrieval buffer (10 mM Tris, 1 mM EDTA solution, and 0.05% Tween 20, pH = 9.0) and incubated at 60 °C overnight (O/N).

The slides were submerged in cold phosphate buffered saline (PBS) for 20 min and permeabilized in 0.5% Triton X-100 in PBS for 10 min at RT. The tissue was then blocked in blocking buffer (2% FBS and 1% bovine albumin serum [BSA] in PBS) for 1 h prior to incubation at 4 °C O/N in a moist chamber with primary antibodies diluted in blocking buffer. The next day, the tissue was incubated with secondary antibodies diluted in blocking buffer at 37 °C for 1 h in a moist chamber. Further, to diminish autofluorescence, the Autofluorescence Quencher Kit was applied to the tissue for 4 min prior to DAPI staining, which was introduced as an intermediate wash. Tissue was washed 3 times in PBS between incubations, rinsed with water, and mounted with Fluoromount mounting medium using precision cover glasses that were sealed with nail polish. The reagents and antibodies used for IHF are listed in the source data in Appendix A.

### 2.6. Microscopy and Image Analysis

#### 2.6.1. Confocal Microscopy

Imaging of FFPE cell blocks for validation of the automated quantification was performed using the point scanning confocal microscope LSM800 (ZEISS, Oberkochen, Germany) with the 40× oil immersion, 1.3 numerical aperture objective, and ZEN Software (ZEISS, Oberkochen, Germany). The image size is 159.73 µm × 159.73 µm with a scaling per pixel of 0.076 µm × 0.076 µm. Images were acquired through channels DAPI (pinhole: 1.00 Airy unit [AU], laser wavelength: 405 nm, laser power: 2%, excitation wavelength: 353 nm, emission wavelength: 465 nm, detector gain: 600 V, and detector offset: −100), Alexa Fluor 568 (pinhole: 1.00 AU, laser wavelength: 561 nm, laser power: 2%, excitation wavelength: 577 nm, emission wavelength: 603 nm, detector gain: 500 V, and detector offset: −200), and Alexa Fluor 647 (pinhole: 1.00 AU, laser wavelength: 640 nm, laser power: 2%, excitation wavelength: 653 nm, emission wavelength: 668 nm, detector gain: 550 V, and detector offset: −100). Output files (.czi, uncompressed) were exported as TIFF files using the ZEISS Zen software (3.8) “Image Export” processing module (LZW compression). The files were subsequently processed using Adobe Photoshop (26.1.0). No intensity normalization or background subtraction was applied to the images.

#### 2.6.2. High-Throughput Fluorescence Microscopy

Quantitative slide scanning of the TMA was carried out using the Axioscan 7 (ZEISS, Oberkochen, Germany) using a 40× air, 0.95 numerical aperture objective. Images were acquired in an automated fashion. Preview imaging of the slide was performed in colour mode RGB with an exposure time of 8.5 milliseconds (ms) using the preview cam. Sample detection was established manually for TMA cores. Automated focus was set up through the DAPI channel—initially through 10× coarse focusing (light source: LED-module 385 nm, light source intensity: 20%, and exposure time: 5 ms) with default quality and sampling (range: 400 µm and step size: 3.25 µm) and smart search. Subsequently, 40× fine focusing (light source: LED-module 385 nm, light source intensity: 5.0%, and exposure time: 20 ms) with default quality and sampling (range: 100 µm and step size: 0.73 µm) and smart search was applied. Both coarse and fine focus were established with onion skin settings for the focus map. The image size is 11.93 mm × 8.30 mm per TMA core with a scaling per pixel of 0.173 µm × 0.173 µm. Images were acquired through channels: DAPI (light source: LED-module 385 nm, light source intensity: 20%, illumination wavelength: 370–400 nm, filter emission wavelength: 412–438 nm, excitation wavelength: 353 nm, emission wavelength: 465 nm, exposure time: 4 ms, depth of focus: 1.03 µm, and binning mode: 2.2), Alexa Fluor 568 (light source: LED-module 567 nm, light source intensity: 70%, illumination wavelength: 540–570 nm, filter emission wavelength: 583–601 nm, excitation wavelength: 577 nm, emission wavelength: 603 nm, exposure time: 300 ms, depth of focus: 1.34 µm, and binning mode: 2.2), Alexa Fluor 647 (light source: LED-module 630 nm, light source intensity: 60%, illumination wavelength: 615–648 nm, filter emission wavelength: 662–700 nm, excitation wavelength: 653 nm, emission wavelength: 668 nm, exposure time: 150 ms, depth of focus: 1.48 µm, and binning mode: 2.2), and Alexa Fluor 750 (light source: LED-module 630 nm, light source intensity: 70%, illumination wavelength: 615–648 nm, filter emission wavelength: 770–800 nm, excitation wavelength: 752 nm, emission wavelength: 779 nm, exposure time: 400 ms, depth of focus: 1.73 µm, and binning mode: 2.2). Output files (.czi, JpegXr compression) were exported as TIFF files using the ZEISS Zen software (3.8) “Image Export” processing module (LZW compression). The files were subsequently analysed using the ZEISS arivis Pro software (4.3.0). Intensities of representative images were adjusted individually for tissues from each of the different organs due to high variation in signal intensities. No intensity normalization or background subtraction was applied to the images.

#### 2.6.3. Manual Analysis

Manual quantification of the FFPE cell blocks was performed using the Adobe Photoshop (26.1.0) count tool. Initially, the total number of cells was counted (only epithelial cells were counted in the tissues), whereafter cells with at least one stressed nucleolus were quantified as nucleolar foci positive. Cells were only scored as positive for nucleolar foci if the number of foci per nucleolus was less than 5, reflecting the expected amount of rDNA clusters within each nucleolus [21,22,23,24].

#### 2.6.4. Automated Analysis

Image segmentation and analysis were performed using the ZEISS arivis Pro (formerly known as Vision4D) software (4.3.0). Importantly, the operations of the analysis software are divided into four categories: image processing, segmentation, use of objects, and export. For the software to function, the order of modules must follow the order of the operation categories. For our setup, the following pipeline was developed for image analysis:

First, an enhancement filter was applied to the images using the “Enhancement Filter” module, applying a Richardson-Lucy deconvolution filter on each of the channels (DAPI, AF568, AF647, and AF750). Next, the dark spheres were enhanced in the RNA Pol II channel, due to its low intensity in the nucleolus, using the “Shape Detection” module (object shape: sphere, object type: dark, object size: 3–15 µm, and sensitivity: 1133). If filtering was applied to segment regions of interest based on the epithelial cell marker, this step was implemented here using the “Intensity Threshold Segmentation” module. Filtering steps are described in more detail in the high-throughput data filtering section. The DAPI stain was then used to detect nuclei using the “Cellpose-based Segmentation” [25] module (Cellpose model: CP, cell diameter: 10 µm, and minimum area: 41,994 nm^2^), and subsequently dark holes (nucleoli) were segmented through the dark spheres with the “Blob Finder” module (diameter: 3 µm, probability threshold: 5.03%, split sensitivity: 65.23% and normalization: first time point). Foci were similarly detected using the “Blob Finder” module (diameter: 0.7 µm, probability threshold: 13%, split sensitivity: 65%, and normalization: first time point). The detected nuclei and nucleoli were filtered to remove artefacts based on size. Nuclei were further filtered to exclude nuclei without overlap with nucleoli. Nuclei touching the border of the image were removed from the analysis. Details of the filtering steps are described in the high-throughput data filtering section. For the TMA, holes in the regions of interest based on the intensity threshold of the pan-cytokeratin staining were filled using the “Segment Morphology” module (method: fill inclusions, perform plane-wise). This step was not implemented for the FFPE cell block analysis. Nucleoli were expanded by 1 pixel using the “Segment Morphology” module (method: dilate objects, pixels: 1, shape: box, perform plane-wise) to allow detection of foci at the nucleolar periphery and interior. Foci were then filtered based on nucleolar localization using the “Compartments” module, with nucleolar foci identified as being within nucleoli or intersecting with 10%. Nucleolar foci were further filtered based on intensity and the number of foci per nucleolus, described in more detail in the high-throughput data filtering section. The nucleoplasm was additionally segmented using the “Object Math” module (Subtract [A–B]), subtracting nucleoli (objects B) from filtered nuclei (objects A). Lastly, cells with nucleolar stress were detected using the “Compartments” module (inputs: filtered nuclei ↳ nucleoplasm ↳ filtered nucleoli ↳ filtered nucleolar foci), relating the filtered foci to nucleoli and nuclei. The automated analysis concurrently measures signal intensities in the nucleoplasm and nucleoli. Object features, including Id, nucleolar intensities (mean and sum), nucleolar area (mean), nucleolar count per nucleus, nucleoplasmic intensities (mean and sum), foci intensities (mean and sum), and nucleolar foci count, were extracted for each filtered nucleus using the “Export Objects Features” module (inputs: filtered nuclei, method: single table, features: as described). Data results are exported as Excel tables (.xlsx) for each image.

#### 2.6.5. High-Throughput Data Filtering

Analysis of the multi-organ TMA included epithelial and carcinoma cells filtering based on the pan-cytokeratin stain [26,27] using the “Intensity Threshold Segmentation” module (method: simple, object type: bright, and threshold: 500). This filtering step was applied in the pipeline after the shape detection (dark spheres). Moreover, the areas of interest based on pan-cytokeratin were filtered based on size (>15 µm^2^), using the “Object Feature Filter” module. Epithelial cells were identified through the “Compartments” relating nuclei to regions of interest with at least 50% intersection. These steps were not implemented for analysis of the FFPE cell blocks and were applied in the pipeline after filling inclusions in the pan-cytokeratin intensity-threshold detection.

Nucleoli were filtered based on size (surface area: >3 µm^2^) using the “Object Feature Filter” module. This step was applied after foci detection. Additionally, the epithelial cells were further filtered in the automated analysis based on size and intensity (surface area: < 400 µm^2^ and sum [intensities #1]: >50,000) or excluded if no nucleoli were identified within the cells using the “Compartments” module (inside or 95% intersection). This step was implemented to exclude samples where the sub-nuclear compartments could not be segmented to remove false positive and negative results in the analysis. Moreover, nuclei touching the border of the image (X left, X right, Y top, and Y bottom) were excluded using the “Touching Edge Filter”. These filtering steps were applied in the pipeline after the filtering of nucleoli.

Lastly, data filtering was performed based on the nucleolar foci count. For this study, the nucleolus was used as an example for studying protein foci accumulation in a sub-cellular compartment. Foci filtering was based on intensity and number (max intensities #2: > 400, #children ≥ 1, and #children ≤ 5).

### 2.7. Experimental Repetitions and Statistics

Validation experiments were performed three times. The multi-organ TMA contained technical duplicates of all tissues. Immunofluorescent staining and analysis were performed once. Error bars represent the standard deviation (SD) in all figures. For the FFPE cell blocks, statistical analysis was performed using an unpaired two-sided student’s t-test with Welch’s correction on data following a Gaussian distribution. Statistical analysis was performed using a Mann–Whitney U test when data were not following a Gaussian distribution. Datasets of multi-organ tissues were tested for Gaussian distribution, and comparison between manual and automated quantification was performed using a two-way ANOVA with Bonferroni correction. Statistical significance in the figures is depicted with stars (* *p* < 0.05, ** *p* < 0.01, *** *p* < 0.001), and no legend shows no statistically significant differences. All statistical analyses were performed in GraphPad Prism (10.4.2).

## 3. Results

### 3.1. Generation of FFPE Cell Blocks from Cancer Cells with Targeted Damage to the Nucleolus

Initially, we set up a tissue-like model in which we could test and verify our method, requiring positive and negative controls of protein distribution patterns in the sub-nuclear compartment of interest for the analysis. For this purpose, we generated FFPE cell blocks from human osteosarcoma, U2OS cells. For the analysis of protein distribution in sub-nuclear compartments in tissue, we used the nucleolus as an example. Changes in sub-nuclear protein accumulation can be induced in the nucleolus by DNA damage due to altered transcriptional activity and translocation of ribosomal and nucleolar proteins either to the nucleolar periphery or the nucleoplasm [28,29,30,31,32]. We took advantage of CRISPR-Cas9 technology to induce DNA damage specifically in the ribosomal DNA (rDNA) genes, which cluster in the nucleolus. This was achieved using the Cas9 endonuclease, either with an rDNA-targeted guide RNA (Cas9-gRNA) or a non-targeting guide RNA as a negative control (Cas9-negative).

**Figure 1:**
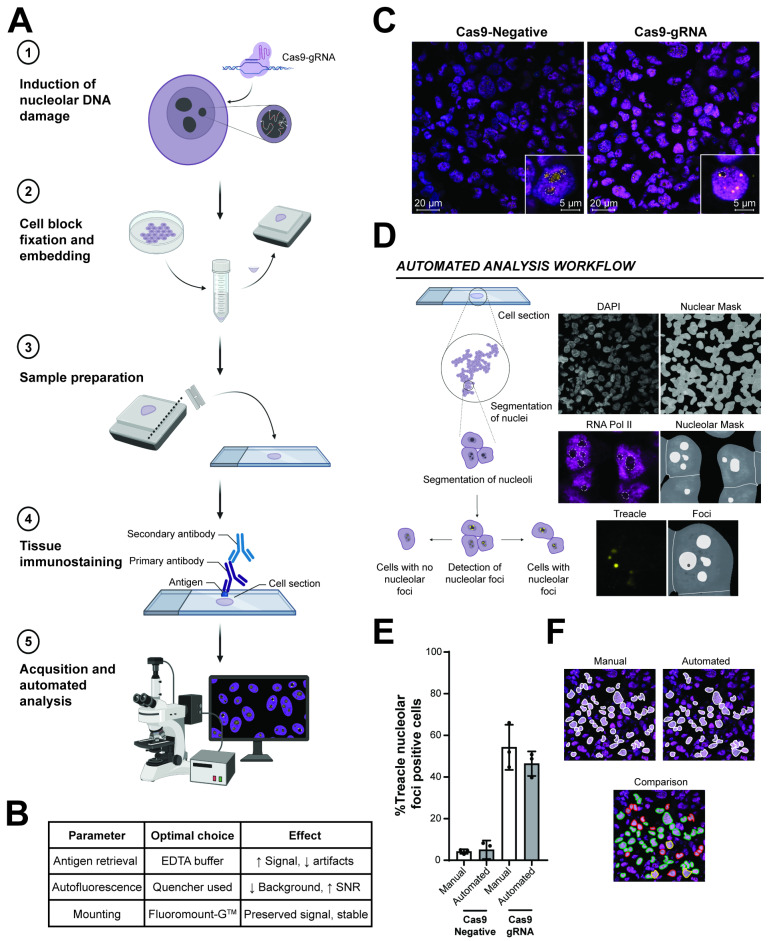
Validation of the automated analysis pipeline in formalin-fixed paraffin-embedded cell blocks generated from U2OS cells with targeted rDNA damage. (**A**) Graphical representation of the workflow for generating formalin-fixed paraffin-embedded cell blocks. The human osteosarcoma U2OS cell line was transfected with Cas9-gRNA to induce targeted damage to the nucleolus using CRISPR-Cas9. The cells with targeted damage were fixed and paraffin-embedded following similar protocols to how human tissue is processed. The generated paraffin-embedded cell blocks were sliced into 4 µm sections, on which tissue immunostaining was performed. Lastly, stained samples were subjected to acquisition and automated analysis. (**B**) Table summarizing the key technical improvements performed for this study. These steps include optimization of antigen retrieval buffer, autofluorescence quenching and mounting medium. (**C**) Representative confocal images of the FFPE cell blocks with targeted rDNA damage (Cas9-gRNA) and without (Cas9-Negative) 8 h after gRNA transfection. Nuclear staining is indicated by DAPI (blue), nucleoplasmic staining is RNA Pol II (magenta), and nucleolar protein staining is Treacle (yellow). Nucleoli are outlined with white dashed lines. Scale bar 20 µm and 5 µm. (**D**) ZEISS arivis Pro (4.3.0) automated analysis workflow of the artificial tissue. First, the analysis segments nuclei using the DAPI staining. Subsequently, sub-nuclear compartments (nucleoli) are identified as dark holes within the nuclei based on the lower signal intensities of RNA Pol II in the nucleolus. Lastly, Treacle foci are quantified within the nucleolus to provide a quantitative measurement of cells positive and negative for nucleolar Treacle foci. (**E**) Representative comparison between nucleolar foci-positive cells (white outline) in Cas9-gRNA treated cells identified by manual (left, top panel) and automated (right, top panel) analysis. The bottom panel indicates the foci-positive cells identified by both manual and automated quantification (green), only by the automated analysis pipeline (yellow), and only by manual quantification (red). (**F**) Comparison between manual and automated quantification of the percentage of cells displaying at least one Treacle foci per nucleolus in the formalin-fixed paraffin-embedded cell blocks. Targeted nucleolar damage was induced in the U2OS cells for 8 h prior to formalin fixation and histoprocessing. The graph depicts mean values ± SD (*n* > 65 cells/experiment). Three biological replicates were performed for the staining and automated analysis. For statistical analysis to compare manual and automated quantification, a two-sided student’s t-test with Welch’s correction was applied.

Once the targeted damage was induced, the U2OS cells were pelleted, fixed, and processed identically to standard operating procedures (SOPs) of how tissue would be processed following surgical removal [33]. The samples were sliced into 4 µm sections that we further subjected to IHF staining. A graphical representation of the workflow for generating the FFPE cell blocks is depicted in Figure 1A.

### 3.2. High-Throughput Detection of the Sub-Nuclear Distribution of the Nucleolar Protein Treacle in FFPE Cell Blocks

We next focused on optimizations of the IHF technique to ensure reproducibility and accuracy of the staining. In this context, we found that the choice of antigen retrieval buffer was very important for the staining quality and signal intensity as well as the introduction of artefacts. We observed a significant improvement in the signal-to-noise ratio in samples submerged in EDTA antigen retrieval buffer compared to citrate buffer. Moreover, we found that implementation of an autofluorescence quencher significantly further improved the signal-to-noise ratios as it reduced background fluorescence through its binding to fluorescent tissue elements such as collagen and blood cells [34,35,36]. The choice of mounting medium also impacted the signal intensity, especially of the far-red channels, where we found that use of specific mounting mediums, i.e., VectaMount^®^, completely removed the signal of the 750 nm excited fluorophores. Instead, we found that using Fluoromount-G^TM^ mounting medium reduced fluorophore quenching during fluorescence microscopy and resulted in good signal intensities even following prolonged storage of the slides at 4 °C. The key technical improvements for the method are summarized in Figure 1B. Following these optimization steps, we found the IHF technique to be of comparable quality in the FFPE cell blocks to that of cultured cells, based on the similarity of staining patterns.

To enable segmentation of the sub-cellular compartments, spatial distribution, and foci accumulation of nucleolar proteins, we used DAPI for detection of nuclei and RNA polymerase II (RNA Pol II) to create an inverse mask for nucleoli based on its low abundance in the nucleolus causing the appearance of dark holes in the staining. RNA Pol II provides a consistent mask for nucleolar identification, as its nuclear distribution is generally not affected by nucleolar stress [19]. Lastly, we used the nucleolar protein Treacle that changes cellular distribution and accumulates in foci upon nucleolar damage [28,29,32,37] to measure changes in sub-nuclear protein distribution (Figure 1C). We detected the markers in the FFPE cell blocks using confocal microscopy and set up an automated analysis using the ZEISS arivis Pro image analysis software (4.3.0) (Figure 1D). In the automated analysis, the nuclei were detected through a module that utilizes the deep-learning-based segmentation algorithm CellPose [25]. The implementation of the open-source CellPose algorithm in the analysis greatly improved segmentation of nuclei in tissues or tissue-like settings where the cell density is high and may overlap or layer to form organized structures. Nucleoli were detected through the RNA Pol II staining, where dark spheres were initially enhanced and subsequently identified within the segmented nuclei (at least 95% overlap). This then enabled us to automate the quantification of protein distribution and foci accumulation within the nucleolus. Here, the changes in protein distribution to foci were filtered based on intensity and the number of foci per nucleolus (maximum 5) corresponding to the expected maximum of rDNA clusters likely to be present within one nucleolus [21,22,23,24]. The output of the analysis provided signal intensity measurements, counts of nuclei, nucleoli and foci, and the number of cells that were positive for nucleolar foci.

To validate our automated quantification, we manually quantified the percentage of cells in the FFPE cell blocks with changes in protein distribution patterns in the nucleolus. We could then assess the accuracy of the automated analysis by comparing the foci-positive cells detected in each experiment by manual quantification to those identified by the automated quantification (Figure 1E). The automated pipeline had an overall accuracy of 82.62% when defining true positives/negatives as those identified through manual quantification. In most cases, the discrepancies between the manual and automated analysis arose from the automated analysis not identifying nucleoli from the RNA Pol II staining, leading to the cells being excluded from the analysis. Moreover, discrepancies sometimes came from the automated analysis inaccurately identifying some cells with intense but normal nucleolar Treacle distribution as foci positive, although this occurred to a lesser extent. However, the results obtained from manual and automated quantification were comparable, and no statistically significant difference was found between the two analyses (Figure 1F), indicating that the variations did not significantly impact the overall results of the automated analysis when compared to the manual quantification.

### 3.3. Epithelial-Specific Detection of Nucleolar Protein Distribution in Tissue

**Figure 2:**
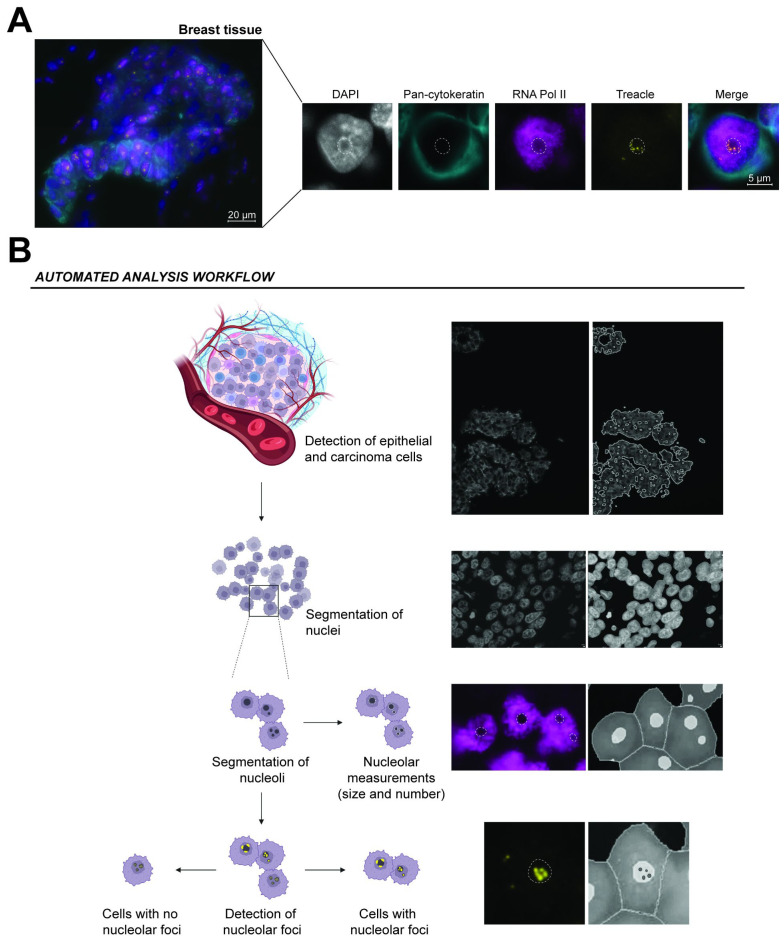
Epithelial cell-specific automated analysis workflow of IHF staining of breast tissue. (**A**) Representative image of IHF staining of breast tissue. Images of tissues were acquired through automated acquisition using the ZEISS Axioscan 7. Nuclei are stained using DAPI (blue), pan-cytokeratin (turquoise) implemented as a marker for epithelial cells, RNA Pol II (magenta) as an inverse marker for nucleoli, and the nucleolar protein Treacle (yellow) as a marker for protein distribution in the nucleolus. Nucleoli are outlined in white dashed lines. Scale bar 20 µm and 5 µm. (**B**) Automated analysis workflow using the ZEISS arivis Pro software (4.3.0). Regions of interest based on the epithelial cell-specific marker are segmented based on intensity threshold. Nuclei are segmented using the deep learning algorithm CellPose within the regions of interest. The nucleoli are segmented within the nuclei through recognition of dark holes in the RNA Pol II staining, and lastly, Treacle foci are identified within the nucleoli. The automated analysis pipeline provides quantitative measures of signal intensities, object sizes, object counts, and nuclei positive for nucleolar protein foci.

Tissue composition had to be considered to successfully transfer our IHF staining and automated analysis from FFPE cell blocks into human tissue samples. Since 80–90% of human malignancies originate from epithelial cells [38], we included an epithelial cell marker when transferring the staining into tissues. This step allowed selective analysis of the epithelial cells in a biopsy from, for instance, a carcinoma and excluded other cell types found in tissues, such as stromal cells. For this purpose, we chose pan-cytokeratin, as it detects a broad spectrum of keratin proteins uniquely expressed in epithelial cell types, irrespective of their tissues of origin [26,27]. In human breast tissues, this resulted in cytoplasmic pan-cytokeratin staining of epithelial cells, nuclei through the DAPI staining, nucleoplasmic staining of RNA Pol II, and lastly a general nucleolar staining of the nucleolar protein Treacle (Figure 2A). Intensity-based identification of regions of interest based on the cytoplasmic pan-cytokeratin staining could then be implemented into the automated analysis, prior to the established segmentation steps, for an epithelial cell-specific analysis (Figure 2B). In the breast tissue, we further observed an overlap of the nucleolar Treacle staining with the dark holes in the RNA Pol II staining that supported the specificity of using RNA Pol II as a reverse mask for nucleoli in tissue. These results demonstrated the use of FFPE cell blocks to develop tissue staining techniques, as the optimizations performed could be directly transferred to human tissues.

### 3.4. High-Throughput Analysis Across Multiple Tissue Types

**Figure 3:**
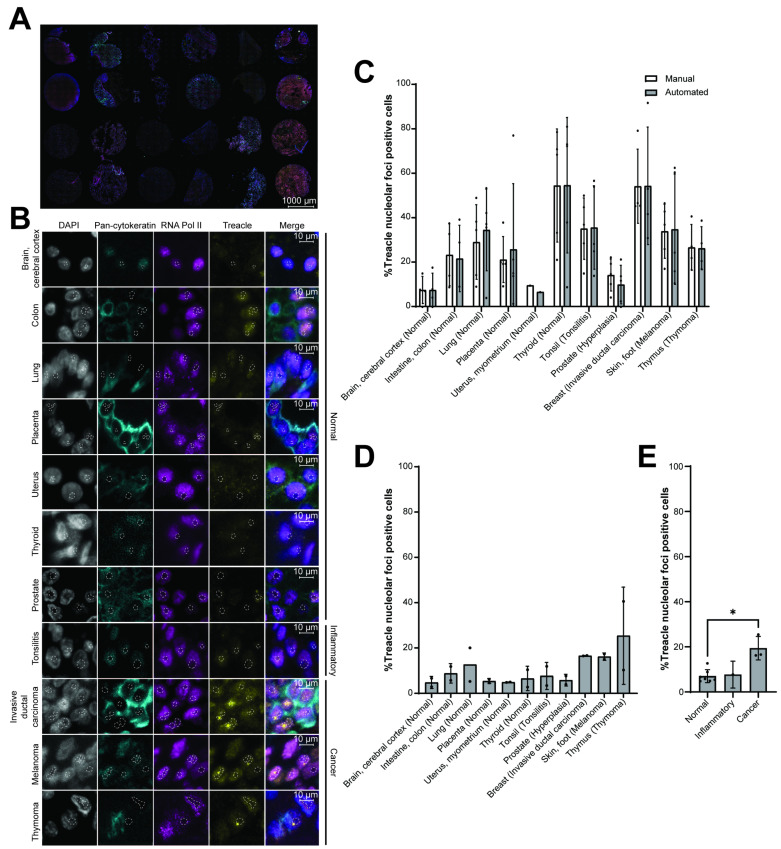
High-throughput automated analysis of epithelial cell-specific IHF staining of multi-organ tissue microarray. (**A**) Representative image of multi-organ tissue microarray acquired through automated acquisition using the ZEISS Axioscan 7. The tissue microarray consists of multiple normal/disease tissues from 12 anatomic sites in duplicates. For representative purposes, intensities were adjusted individually for each tissue core. Scale bar 1000 µm. (**B**) Representative images of the different organ tissues on the tissue microarray. Tissue from the liver (viral hepatitis-related cirrhosis) was excluded from the representative images, as the RNA Pol II signal was too weak, not allowing the segmentation of nucleoli. The tissues have been separated into groups of normal, inflammatory, or cancer tissues. Nuclei are stained using DAPI (blue) and pan-cytokeratin (turquoise) to mask the cytoplasm of epithelial cells and RNA Pol II (magenta) to stain the nucleoplasm, enabling visualization of nucleoli as dark holes in the staining. The nucleolar protein Treacle (yellow) is a marker for the protein of interest to quantify protein foci. Nucleoli are outlined in white dashed lines. Scale bar 10 µm. (**C**) Comparison between the manual and automated quantification of the percentage of epithelial cells displaying at least one Treacle foci per nucleolus in randomly selected regions of each of the multi-organ tissues in the TMA. The graph depicts mean values ± SD (*n* > 100 cells/region of interest). Quantifications of each region of interest are depicted as individual points. For statistical analysis to compare manual and automated quantification, a two-way ANOVA with Bonferroni correction was applied. (**D**) Percentage of epithelial cells displaying at least one nucleolar Treacle foci in the multi-organ tissue microarray obtained from the high-throughput analysis. Multi-organ tissues are separated into tissue types, each displaying a percentage of cells with nucleolar Treacle accumulation. The graph depicts mean values ± SD (*n* = 100–10,443 cells/tissue core). One TMA with technical duplicates was analysed, and each duplicate is depicted as an individual point. (**E**) Percentage of epithelial cells displaying at least one nucleolar Treacle foci in the multi-organ tissue microarray obtained from the high-throughput analysis. Multi-organ tissues are separated into groups of normal, inflammatory, or cancer tissues in which to compare the percentage of cells with nucleolar Treacle accumulation. The graph depicts mean values ± SD (*n* = 100–10,443 cells/tissue core). One TMA with technical duplicates was analysed. For statistical analysis to compare the percentage of epithelial cells with nucleolar Treacle foci between normal (*n* = 7 patients) and cancer (*n* = 3 patients), a two-sided Mann–Whitney U test was applied. Statistical significance is depicted with stars (* *p* < 0.05, ** *p* < 0.01, *** *p* < 0.001), and no legend shows no statistically significant differences.

To implement automated imaging of tissue samples, we used the ZEISS Axioscan 7. The ZEISS Axioscan 7 is a high-performance slide scanner that can acquire high-resolution images of tissue samples in an automated manner in up to 9 fluorescent channels. This enables high-throughput imaging of the sample and scalability regarding the number of samples (Figure 3A).

To test the robustness of our staining approach between tissue types, we took advantage of a tissue microarray (TMA) consisting of multiple normal/disease tissues from 12 anatomic sites. We found that the staining steps included in the protocol transferred well across different tissue types (Figure 3B).

To verify the ability of the automated analysis to accurately identify cells with changes in nucleolar Treacle distribution, we randomly selected up to five regions of interest (combined containing more than 100 epithelial cells) within each tissue type to compare the results obtained from manual quantification to the automated analysis output. The results from the automated pipeline were comparable to manual quantification, with no statistically significant differences (Figure 3C). However, we observed some variation between the quantification of individual images, particularly in lung, placenta, liver, breast, and skin tissues (Supplementary Figure S1A). When we explored the underlying reason, most discrepancies, again, arose from limitations related to nucleolar segmentation from the RNA Pol II signal in the automated analysis (Supplementary Figure S1B). However, despite these occasional discrepancies, the variations had minimal impact on the overall result.

Analysis of only a few sections of the tissues may not capture the full variability of the tissue, potentially leading to non-representative results, whereas high-throughput image analysis can capture the inherent heterogeneity of tissue samples [39]. We thus proceeded to apply the automated analysis pipeline to the entire TMA to assess changes in Treacle distribution across all multi-organ tissues. Notably, we observed quite high variability between the results obtained from the analysis of randomly selected regions (Figure 3C) and the high-throughput analysis of the entire TMA (Figure 3D). For instance, analysis of only a small section of the thyroid tissue revealed around 54% of epithelial cells with changes in nucleolar Treacle distribution, whereas only 6.5% of epithelial cells in the same thyroid tissue had changes in the nucleolar Treacle distribution when analysing the full tissue section. This variability was observed between the outputs for most of the tissues, including intestine, lung, placenta, thyroid, tonsil, breast, and skin tissues, and emphasizes the need for high-throughput analysis to capture the full heterogeneity associated with tissues.

Since nucleolar functions are known to be deregulated in cancer [40], we asked whether the pipeline could detect a change in the presence of nucleolar Treacle protein foci. The analysis showed a significant increase in the percentage of epithelial cells with nucleolar Treacle foci in the cancer tissues compared to normal tissues (Figure 3E). One inflammatory tissue sample was analysed but did not show an apparent increase compared to the normal tissue; however, no statistical conclusions can be drawn from just a single patient. These findings demonstrated how the presented method allows investigation of known cellular phenotypes in tissues to enable further investigation of their clinical relevance. With the development of the IHF staining technique, automated high-content imaging, and automated AI-based image analysis tools, we provide a scalable pipeline for investigation of sub-nuclear protein distributions in tissues.

## 4. Discussion

In this study, we present a tissue-specific IHF staining as well as subsequent semi-automated acquisition and analysis to provide a high-throughput tool for identification of protein distribution in sub-nuclear compartments. This method provides a tool that can benefit molecular research efforts and clinicians, as it can provide detailed analysis of protein behaviour in an individual patient’s tissues.

In this study, our semi-automated analysis approach quantified the redistribution of the nucleolar protein Treacle within epithelial cells. While recent advancements in multiplexed immunofluorescence have enabled the simultaneous detection of 30–60 markers within a single tissue section [9], the primary focus of many of these methods has been on cell-type classification [41,42], expression profiling [43], or spatial transcriptomics [44]. This is accomplished by several multiplexing methods, i.e., co-detection by indexing (CODEX) [45,46,47], multiplexed ion beam imaging (MIBI) [48,49,50], and imaging mass cytometry [51,52], which have been developed over recent years for cellular-level human tissue analysis. However, these methods are typically optimized for broadscale marker detection [51,53], and detailed analysis of sub-cellular structures or protein foci formation remains underexplored in many of these applications. IHF provides an alternative approach for detection of protein distribution within sub-nuclear compartments. The IHF method is currently limited to nine markers but does enable the combination of multiple markers of interest. The focus on protein localization and spatial distribution makes it particularly suitable for studies focused on protein phase separation, aggregation pathology, or other fine-scale spatial events. Furthermore, in contrast to many multiplex platforms, our approach builds on conventional IF methods commonly used in in vitro studies, enabling extension of cell culture findings into human tissues with minimal adaptation in antibody selection.

The automated analysis pipeline for assessing changes in nucleolar Treacle distribution showed a promising accuracy of 82.62% compared to manual quantification of the FFPE cell blocks; however, it does have some limitations. One issue is the architecture of tissues where cells are in different focal planes, impacting the accuracy of the automated method in identifying nucleoli from the RNA Pol II staining. This can lead to inaccuracies in the identification of nucleoli, as the automated pipeline cannot currently adjust for variations in cellular layering. Thus, further development of the image acquisition, such as 3D imaging or focal stacking [54], could improve the analysis across cell layers, allowing for more accurate identification of nucleoli. Implementation of a second nucleolar marker could also enhance the ability of the pipeline to identify nucleoli in individual cells, thereby improving accuracy. To address variation associated with tissue heterogeneity, it would be informative to further develop the analysis pipeline to provide spatial information not only at the cellular but also at the tissue level. Moreover, while pan-cytokeratin is a robust and widely used epithelial cell marker, a limitation of this study lies in its inability to distinguish between normal epithelial cells and carcinoma within cancerous tissues. This limitation is particularly relevant in the context of heterogenous tumour tissues, where both normal epithelial and carcinoma coexist [55]. Incorporation of additional markers such as Ki67 [56] or specific cytokeratin subtypes (i.e., CK5/6, CK7, and CK20) [57,58] could improve the identification of epithelial subpopulations and distinguish carcinoma cells more accurately from their normal counterparts. The robustness across varying tissue types will also have to be assessed further as specific applications are developed. Despite these challenges, the performance of the automated analysis is comparable to manual quantification, supporting its potential for further applications.

The potential applications of the IHF method can be extended depending on the question of interest by replacing the specific markers used for these targets in the IHF technique. Moreover, the automated analysis does not need to be confined to sub-nuclear compartments. Changes in distribution of nuclear proteins without segmentation of sub-nuclear compartments may be sufficient for use in some translational and clinical applications. FFPE cell blocks can also be used as a tool to test the specificity of antibodies before applying them to human tissue samples.

An increased examination of cellular phenotypes in a tissue context can be clinically important as the distribution of proteins plays central roles in various diseases, including cancer, neurodegeneration, and ageing-related conditions [59,60,61]. The method could potentially improve stratification of patients for targeted therapies based on information derived from protein distribution. For instance, in the context of homologous recombination deficiency (HRD), RAD51 foci can guide the use of PARP inhibitors (PARPis) in patients without mutations in HR-related genes suffering from breast and ovarian cancer [62,63,64]. Currently mutational signatures are used to identify HRD patients, but the genetic profile presents the history of the tumour and may not reflect the actual tumour status in case of reversion mutations. Optimizing the detection of nuclear RAD51 foci could thus provide a functional readout of HR status and guide treatment decisions in cases where genomic data alone are insufficient. Moreover, this method could also serve as a platform to complement current staining and analysis methods for classification of HER2 breast cancer patients that are currently performed using IHC and in situ hybridization (ISH) scoring to distinguish HER2 positive and negative patients [65,66,67,68]. The increasing number of HER2-targeting compounds has raised the question of whether patients with low HER2 expression should be categorized separately, as they may also derive benefit from HER2 targeting. However, this would require more accurate measurements of HER2 levels that could be achieved using IHF [69]. Recently HER2 isoforms and sub-cellular localization were also demonstrated to change in response to treatment and may explain required resistance to trastuzumab [70]. Analysis of the sub-cellular distribution of HER2 (membrane/cytoplasm/nuclei) may therefore provide additional information of clinical value, and such information could be obtained through IHF analysis. This also applies to other therapeutic biomarker stainings currently performed using IHC, such as PDL-1. The current PDL-1 scoring is complex, with membrane positivity being crucial in tumour cells, whereas either membrane or cytoplasmic staining is scored positively for tumour-infiltrating cells [71]. Automated acquisition and analysis could be valuable tools in this context. Furthermore, IHF could enable the development of PDL-1 detection assays beyond the commercial assays currently used [71]. While future efforts are needed to implement and optimize the antibodies for the IHF technique for these purposes, the improved approach to detect protein distributions of current and future biomarkers holds great promise for refining patient stratification, enabling more precise and personalized treatment strategies compared to the conventional techniques.

Beyond oncology, this high-throughput approach also holds promise in neurodegenerative diseases, where protein aggregates like tau in Alzheimer’s [72] or α-synuclein in Parkinson’s disease [73,74] have been shown to play central roles in disease initiation and progression, with ongoing research investigating their potential as biomarkers for early diagnosis and disease monitoring [75,76,77,78].

AI image analysis has significantly improved in recent years, offering a platform to process and analyse vast amounts of data. Building on these capabilities, AI could further be implemented in our method to develop unbiased recognition of cellular protein distribution patterns by training machine learning models on tissue samples with known alterations in protein distribution for improved recognition.

Altogether, the IHF method provides a high-throughput tool to improve our current understanding of protein status in their native tissue environment. It holds the potential to extend the identification of prognostic and predictive biomarkers for disease treatment by bridging the gap in translation of in vitro findings into tissue-based systems, enabling the investigation of protein behaviour in more physiologically relevant contexts.

## Data Availability

The automated analysis ZEISS arivis Pro 4.3.0 pipeline and original microscopy images of the data are available upon request. All other data supporting the findings of this study are included in the main article and associated files. Source data are provided in the Appendix A. Further information and requests for resources and reagents should be directed to and will be fulfilled by the lead contact, Dorthe Helena Larsen (dhl@cancer.dk).

## Supplementary Materials

The following supporting information can be downloaded at: https://www.mdpi.com/article/10.3390/cells14141109/s1, Figure S1: Validation of automated analysis pipeline in multi-organ tissue microarray; Table S1: sgRNA sequences; Appendix A.

## Abbreviations

The following abbreviations are used in this manuscript:

AI	Artificial intelligence
AU	Airy unit
BSA	Bovine albumin serum
CODEX	Co-detection by indexing
DMEM	Dulbecco’s modified Eagle’s medium
DSB	Double-stranded breaks
FBS	Foetal bovine serum
FFPE	Formalin-fixed paraffin-embedded
HRD	Homologous recombination deficiency
IF	Immunofluorescence
IHC	Immunohistochemistry
IHF	ImmunoHistoFluorescence
ISH	In situ hybridization
MIBI	Multiplexed ion beam imaging
ms	Milliseconds
O/N	Overnight
PARPi	Poly(ADP)-ribose polymerase inhibitors
PBS	Phosphate-buffered saline
Pen-strep	Penicillin-streptomycin
rDNA	Ribosomal DNA
RNA Pol II	RNA Polymerase II
RT	Room temperature
SD	Standard deviation
SOP	Standard operating procedure
TMA	Tissue microarray
WT	Wild-type

## Appendix A

**SOURCE DATA**		
**REAGENT or RESOURCE**	**SOURCE**	**IDENTIFIER**
Antibodies		
Treacle	Santa Cruz	Cat# sc-374536; RRID: AB_10987865
RNA polymerase II subunit B1 (phospho-CTD Ser-5) Antibody, clone 3E8	Sigma-Aldrich	Cat# 04-1572-1; RRID: AB_10615822
Pan-cytokeratin	Abcam	Cat# ab234297; RRID: AB_2895302
Goat anti-Mouse IgG (H + L) Highly Cross-Adsorbed Secondary Antibody, Alexa Fluor™ 568	Invitrogen^TM^	Cat# A-11031; RRID: AB_144696
Goat anti-Rat IgG (H + L) Cross-Adsorbed Secondary Antibody, Alexa Fluor™ 647	Invitrogen^TM^	Cat# A-21247; RRID: AB_141778
Goat anti-Rabbit IgG (H + L) Cross-Adsorbed Secondary Antibody, Alexa Fluor™ 750	Invitrogen^TM^	Cat# A-21039; RRID: AB_2535710
Chemicals, peptides, and recombinant proteins		
Dulbecco’s modified Eagle’s medium (DMEM), GlutaMAX^TM^ supplement	Gibco, Life Technologies	Cat# 31966-021
Foetal Bovine Serum (FBS)	Gibco, Life Technologies	Cat# A5256701
Penicillin-Streptomycin (pen-strep)	Gibco, Life Technologies	Cat# 15140-122
TrypLE^TM^ Express	Gibco, Life Technologies	Cat# 12604-013
Opti-MEM^TM^ reduced serum medium, GlutaMAX^TM^ supplement	Gibco, Life Technologies	Cat# 51985-026
Lipofectamine™ CRISPRMAX™ Cas9 Transfection Reagent	Invitrogen^TM^	Cat# CMAX00015
TrueCut^TM^ Cas9 Protein v2	Invitrogen^TM^	Cat# A36499
TrueGuide^TM^ synthetic gRNA negative control, non-targeting 1	Thermo Fisher	Cat# A35526
TrueGuide^TM^ synthetic gRNA	Thermo Fisher	Cat# A35514
Formaldehyde 4% stabilised, buffered	VWR Chemicals	Cat# 9713.1000
Ethanol 96% vol	VWR Chemicals	Cat# 83804.360
Ethanol absolute	VWR Chemicals	Cat# 83813-360
Xylene	VWR Chemicals	Cat# 28973.294
Cellsafe plus, biopsy cassettes	Hounisen	Cat# 2270.5060
Paraffin	Hounisen	Cat# 2270-6060
VWR^®^ SuperFrost^®^ Plus, Adhesion Slides	VWR Chemicals	Cat# 631-0108
VECTABOND^®^ reagent, tissue section adhesion	Vector Laboratories	Cat# SP-1800-7
Tris ultrapure	VWR Chemicals	Cat# 0497
Ethylenediaminetetraacetic acid (EDTA, pH = 8.0)	Sigma-Aldrich	Cat# E9884
Tween^®^ 20	PanReac Applichem	Cat# A1389
Triton^TM^ X-100	Sigma-Aldrich	Cat# T8787
Bovine serum albumin (BSA)	Sigma-Aldrich	Cat# A7030
Vector^®^ TrueVIEW^®^ Autofluorescence Quenching Kit	Vector Laboratories	Cat# SP-8400-15
DAPI (4’,6-diamidino-2-phenylindole, dihydrochloride)	Invitrogen^TM^	Cat# D1306
Fluoromount-G^TM^ mounting medium	Invitrogen^TM^	Cat# 00-4958-02
Precision cover glass thickness No. 1.5H (tol. ± 5 µm)	Marienfeld	Cat# 0107222
Sequences		
gRNA sequences	Supplementary Information	N/A
Experimental models: Cell lines		
U2OS cell line	ATCC	Cat# HTB-96
Experimental models: Tissue microarrays		
Universal IHC/ISH control tissues, 12 cases (1.5 mm)	Pantomics Inc.	Cat# UNC241
Software and algorithms		
ZEISS Zen 3.8	ZEISS	N/A
ZEISS arivis Pro 4.3.0	ZEISS	N/A
GraphPad Prism 10.4.2 for Windows	GraphPad	N/A
