# Supplementary material for: A High-Throughput ImmunoHistoFluorescence (IHF) Method for Sub-Nuclear Protein Analysis in Tissue
Source: Cells. 2025 Jul 18;14(14):1109. doi: 10.3390/cells14141109 (PMC12293571; doi:10.3390/cells14141109)
Supplement: Supplementary file 1 [file cells-14-01109-s001.zip › cells-3735074-supplementary.pdf]

# SUPPLEMENTARY INFORMATION

## Supplementary Figure S1

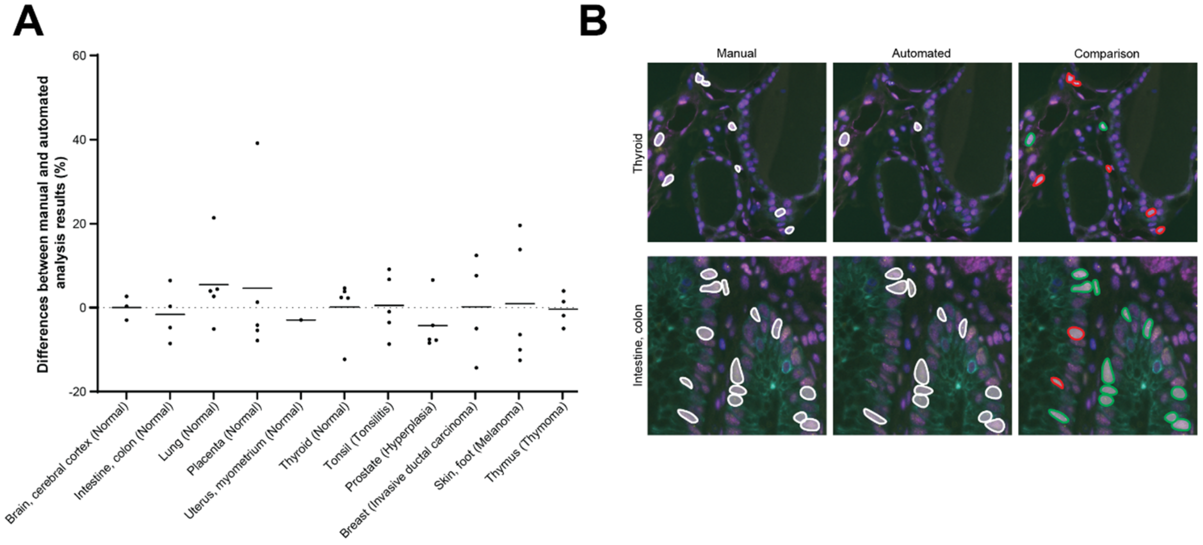


**Supplementary Figure S1. Validation of automated analysis pipeline in multi-organ tissue microarray.** (A) Differences between manual and automated quantification of randomly selected regions of interest in multi-organ tissues. Individual points depict one randomly selected region. The differences are calculated by subtracting the results from the manual analysis to the automated results. (B) Representative comparison between nucleolar foci positive cells (white outline) in thyroid (top panel) and colon (bottom panel) tissues. Foci positive cells identified by manual (left panels) and automated (middle panels) quantification, and a comparison between the results obtained from the two analyses (right panels). The comparison indicates the foci positive cells identified in both the manual and automated analysis (green) and only from the manual analysis (red).

**Supplementary Table S1**

| sgRNA sequences | |
|---|---|
| **sgRNA** | **Sequences 5′-3′** |
| sgRNA1 | CGAGAGAACAGCAGGCCCGC |
| sgRNA3 | GATTTCCAGGGACGGCGCCT |
